# Evidence of altered fatty acid metabolism in dogs with naturally occurring valvular heart disease and congestive heart failure

**DOI:** 10.1007/s11306-022-01887-7

**Published:** 2022-05-30

**Authors:** Jenny Wilshaw, A. Boswood, Y. M. Chang, C. J. Sands, S. Camuzeaux, M. R. Lewis, D. Xia, D. J. Connolly

**Affiliations:** 1 Department of Clinical Science and Services, Royal Veterinary College, University of London, Hawkshead Lane, North Mymms, Hatfield, Hertfordshire, AL9 7TA London, United Kingdom; 2 Research Support Office, Royal Veterinary College, University of London, London, United Kingdom; 3 National Phenome Centre, Department of Metabolism, Digestion and Reproduction, Imperial College London, London, United Kingdom; 4 Department of Comparative Biomedical Science, Royal Veterinary College, University of London, London, United Kingdom

## Abstract

**Introduction:**

Myxomatous mitral valve disease (MMVD) is the most common cardiac condition in adult dogs. The disease progresses over several years and affected dogs may develop congestive heart failure (HF). Research has shown that myocardial metabolism is altered in cardiac disease, leading to a reduction in β-oxidation of fatty acids and an increased dependence upon glycolysis.

**Objectives:**

This study aimed to evaluate whether a shift in substrate use occurs in canine patients with MMVD; a naturally occurring model of human disease.

**Methods:**

Client-owned dogs were longitudinally evaluated at a research clinic in London, UK and paired serum samples were selected from visits when patients were in ACVIM stage B1: asymptomatic disease without cardiomegaly, and stage C: HF. Samples were processed using ultra-performance liquid chromatography mass spectrometry and lipid profiles were compared using mixed effects models with false discovery rate adjustment. The effect of disease stage was evaluated with patient breed entered as a confounder. Features that significantly differed were screened for selection for annotation efforts using reference databases.

**Results:**

Dogs in HF had altered concentrations of lipid species belonging to several classes previously associated with cardiovascular disease. Concentrations of certain acylcarnitines, phospholipids and sphingomyelins were increased after individuals had developed HF, whilst some ceramides and lysophosphatidylcholines decreased.

**Conclusions:**

The canine metabolome appears to change as MMVD progresses. Findings from this study suggest that in HF myocardial metabolism may be characterised by reduced β-oxidation. This proposed explanation warrants further research.

**Supplementary information:**

The online version contains supplementary material available at 10.1007/s11306-022-01887-7.

## Introduction

The heart is one of the most metabolically active organs in the body, requiring a continuous supply of adenosine triphosphate (ATP). In healthy cardiac tissue the majority of ATP is generated by oxidative phosphorylation within the mitochondria. It is estimated that approximately 70% of ATP is produced as a result of fatty acid (FA) oxidation and alternative substrates, predominantly pyruvate, account for the remainder (Stanley et al., 2005). In cardiovascular disease myocardial metabolism is affected in several ways, including altered substrate selection and a loss of metabolic flexibility. Substrate use varies with disease aetiology and severity, and most studies report a reduction in FA oxidation in heart failure (HF) (Doenst et al., 2013). It is not known when in the course of disease these changes occur, however it is hypothesised that alterations in FA metabolism develop alongside HF (Chandler et al., 2004; Heather et al., 2006). Due to the intrinsic relationships that exist between FA and glucose pathways, concomitant changes in glucose metabolism are also reported causing an increased dependence upon glycolysis for the production of ATP (Doenst et al., 2013). With chronicity, alterations in substrate use are considered maladaptive and may promote hypertrophy and cardiac dysfunction (Beauloye et al., 2011; Chavez & Summers, 2012; Fugio et al., 2020; Gibb & Hill, 2018; Ingwall & Weiss, 2004; MacEyka & Spiegel, 2014; Nakamura & Sadoshima, 2018; Pettus et al., 2002; Schönekess et al., 1996; Wambolt et al., 2000). Evidence in favour of a causal relationship between myocardial metabolism and disease progression also comes from inherited disorders and knockout models where HF develops as a result of disordered metabolism (Abdurrachim et al., 2015; Marín-García & Goldenthal, 2002). Treatments targeting metabolic remodelling are therefore the subject of ongoing research, and several pathways show therapeutic potential (Bersin et al., 1994; Berthiaume et al., 2014; Dabkowski et al., 2013; Lahey et al., 2014; Yamanushi et al., 2014). Furthermore, several studies have found that metabolic “fingerprints” show potential for disease diagnosis, assessment of severity and prognostication (Cheng et al. 2017).

At present, this topic is not well described in naturally occurring animal models of cardiac disease. As the comorbidities and environmental exposures of aging humans are markedly less prevalent in dogs, a canine population could be used to examine the cardiac metabolome with fewer confounding factors. In addition, the canine lifespan is shorter than that of humans, so valuable information encompassing the development and progression of disease can be gathered more efficiently (Greer et al., 2007). Myxomatous mitral valve disease (MMVD) is the most frequently diagnosed cardiac condition in adult dogs, with an estimated prevalence of 3.5% in veterinary practice (UK) (Mattin et al., 2015). Some dogs with MMVD will develop HF which is defined as the presence of cardiogenic pulmonary oedema (Keene et al., 2019). MMVD is routinely monitored using echocardiography and objective measurements of chamber size are used to quantify severity and assist in staging disease using guidelines produced by the American College of Veterinary Internal Medicine (ACVIM), based upon the American College of Cardiology/American Heart Association classification system (Atkins et al., 2009; Hunt et al., 2001; Keene et al., 2019). To date, only three studies have examined the metabolome of dogs with naturally occurring MMVD, producing results that are suggestive of a shift from FA oxidation to glucose dependent pathways (Li et al., 2015, 2020, 2021). It appears that substrate use is altered in MMVD (Li et al., 2015, 2020), and is associated with the development of heart failure (Li et al., 2021). To date, no study has examined whether this shift is appreciable within individual dogs as their disease progresses. As this change is common to other forms of cardiac hypertrophy (Allard et al., 1994; Christe & Rodgers, 1994), the prolonged asymptomatic phase of canine MMVD could be used to evaluate whether intervention can ameliorate maladaptive metabolic alterations prior to the onset of HF.

The aim of this study was to evaluate if the lipid metabolome changes as dogs progress to a more advanced stage of MMVD.

## Methods

### Study Design

The study was a retrospective analysis of residual blood samples. The protocol was approved by the Royal Veterinary College’s ethics and welfare committee (URN 2017 1747-2) and informed written consent was provided by owners to use samples and data for research.

Dogs were client owned and had been longitudinally monitored at a cardiovascular research clinic (2004–2017). Patients underwent biannual examinations by a board-certified veterinary cardiologist (AB) who obtained standard right parasternal echocardiographic views at each visit. Stage B1 defined preclinical dogs where the left atrial to aortic root ratio (LA:Ao) (Hansson et al., 2002) and the normalised left ventricular internal diameter at end diastole (LVIDDN) (Cornell et al., 2004) fell below 1.50 and 1.85 respectively (Atkins et al., 2009). A diagnosis of stage C disease was primarily reliant upon radiographic signs of congestive heart failure (HF) (Keene et al., 2019). In the absence of radiographs, historical and physical examination findings consistent with HF were interpreted alongside echocardiography. At each visit, a jugular blood sample was collected and placed into a serum gel tube (Sarstedt, Nümbrecht, Germany). Samples were stored at 4 °C for up to 5 hours before undergoing centrifugation at 1000 g for 15 minutes. Serum was then removed and stored at -80 °C until the time of analysis.

Dogs that had progressed from ACVIM stage B1 to stage C (HF) were targeted to capture disease progression. Selection criteria were based upon breed, diet, and the availability of stored serum. Cavalier King Charles Spaniels are of interest when studying MMVD because of the breed’s marked predisposition, early disease onset, risk of progression and the degree to which neuroendocrine pathways are activated (Beardow & Buchanan, 1993; Hezzell et al., 2014; López-Alvarez et al., 2014; Mattin et al., 2019). A cohort of CKCS were therefore selected to form the majority breed, facilitating between and within breed comparisons (CKCS/ non-CKCS). Data and stored serum from a patient’s first visit in stage B1 and C were used for analyses.

Paired samples from a separate group of 10 unaffected dogs (ACVIM stage A) dogs were included as a control population. These dogs had been examined at the same research clinic and did not have a heart murmur or other evidence of cardiac disease on cardiothoracic auscultation. Controls were age matched and preferentially selected if the time interval between their paired visits was of a similar length to those of the affected dogs. Cavalier King Charles Spaniels were again selected as the majority breed in this group.

### Analyses

A total of 80 serum samples from 40 animals were analysed using ultra-performance liquid chromatography-mass spectrometry (UPLC-MS) to generate lipidomic global profiling datasets. Serum samples were prepared as previously described (Izzi-Engbeaya et al., 2018) for the separation of lipophilic analytes (e.g., complex and neutral lipids) by reversed-phase chromatography (lipid RPC). Details are provided as an online supplement (Supplementary Methods). Analyses were performed using freeware (R 3.5.1, R Foundation for Statistical Computing, Vienna, Austria). Descriptive statistics for continuous variables are reported as the median (lower quartile, upper quartile) and as a proportion (frequency) for categorical variables. Mann-Whitney U and Fisher’s Exact tests were used to compare descriptive data.

#### Association of molecular features with disease severity

Features acquired using ultra-performance liquid chromatography mass spectrometry (UPLC-MS) underwent probabilistic quotient normalization (Dieterle et al., 2006) and univariate scaling prior to their inclusion in statistical models. Linear mixed-effects models were used to analyse the data from paired visits and separate models were created for each feature. In the analysis of the affected population, each feature was regressed on ACVIM stage, breed and the two-way interaction term for stage and breed. Breeds were grouped as CKCS or non CKCS. Patient case number was entered into models as a random effect to account for any subject specific variance. False discovery rate adjustment was applied to control for type I errors (Efron et al., 2015) and associations between variables were considered significant when the false discovery rate corrected value, Q, was < 0.05. For the analysis of the control population, similar methods were followed, although visit number was entered instead of disease stage in regression models.

To evaluate the consistency of results, orthogonal projection to latent structure discriminant analysis (OPLS-DA) (Trygg & Wold, 2002) was performed for control and affected populations respectively. The validity of OPLS-DA models was assessed by cross-validation (Q2Ŷ values) and permutation testing. Robust models were defined as those with a positive Q2Ŷ, and with < 5% of Q2Ŷ values from 1000 randomly permuted models greater than that of the true value. For each explanatory variable, the weights from OPLS-DA were plotted against the Q*-*values generated by linear mixed effects modelling and visually assessed for agreement.

#### Annotation of metabolites that vary with disease stage

Features that demonstrated significant differences in intensity between disease stage and/or breed groups by mixed-effects modelling (Q < 0.05) were selected for further annotation efforts. After manual review of the feature extraction and deconvolution quality produced by the Progenesis QI software, 69 features were selected as promising candidates for annotation. Spectra were generated using tandem mass spectrometry and compared to in-house, in-silico and online spectral databases (including LIPID MAPS and the Human Metabolome Database) to identify unique lipids and understand their potential relevance in cardiac disease. The change in concentrations of these lipids between visits was assessed for the control and affected populations and plotted on a heatmap for the purpose of comparison.

## Results

### Patient Population

The study population was formed of 30 affected and 10 control dogs. Cavalier King Charles Spaniels comprised 67% (n = 20) of the affected group and 50% (n = 5) of the control group. The affected group otherwise comprised 1 Bichon Frisé, 1 Chihuahua, 1 Cocker Spaniel, 1 Maltese, 2 Shih Tzus, 1 Yorkshire terrier and 3 cross breed dogs. The 5 non-CKCS dogs in the control group were 1 Jack Russell Terrier, 2 Poodles, 1 Pug and 1 Shih Tzu. At the first visit, the median age of controls was 6.25 years (LQ = 5.35, UQ = 8.20) and for cases it was 7.65 years (LQ = 5.85, UQ = 8.93). The median number of days between collection of samples was longer for cases (median time between B1 and C = 1277 days, LQ = 823, UQ = 1579, range = 413–2338) than controls (median time between visits 1 and 2 = 819 days, LQ = 635, UQ = 1211, range = 148–1746). Descriptive statistics at baseline are summarised in Table 1.

**Table 1:** Characteristics of the affected and control populations at their baseline visit

		**Group**		*P*
**Variable**		**Controls (n = 10)**	**Cases (n = 30)**	
Age (years)		6.3 (5.4, 8.2)	7.7 (5.9, 8.9)	0.301
Breed	CKCS	50% (5)	67% (20)	0.457
Days between samples		819 (635, 1211)	1277 (823, 1579)	0.089
Sex	Male entire	0% (0)	23% (7)	0.334
	Male neutered	30% (3)	37% (11)	
	Female entire	10% (1)	3% (1)	
	Female neutered	60% (6)	37% (11)	
Weight (kg)		7.9 (6.6, 10.6)	10.0 (7.4, 11.7)	0.346

Legend: Categorical variables are reported as the proportion (frequency). Continuous variables are reported as the median (lower quartile, upper quartile). Between group comparisons were made using Mann-Whitney U and Fischer’s Exact tests. CKCS, Cavalier King Charles Spaniel

As expected, dogs with MMVD showed evidence of disease progression at their second visit. Measurements of LA:Ao and LVIDDN were significantly greater when dogs were in HF (LA:Ao P < 0.001, LVIDDN P < 0.001) and left ventricular systolic function declined (LVIDSN P < 0.001). Dogs in HF showed weight loss although this difference was not significant (P = 0.065). Weight loss was not observed in control dogs (Visit 1: 7.85 kg, LQ = 6.55, UQ = 10.58. Visit 2: 7.40 kg, LQ = 6.70, UQ = 18.20. P = 0.674). Further information is summarised in Table 2.

**Table 2:** A summary of the clinical characteristics of the affected population at each visit

Variable		Stage B1	Stage C
Age (years)		7.7 (5.9, 8.9)	11.3 (9.1, 12.3)
LA:Ao		1.17 (1.09, 1.28)	1.72 (1.53, 1.97)
LVIDDN		1.60 (1.53, 1.73)	2.21 (2.03, 2.35)
LVIDSN		0.96 (0.88, 1.08)	1.09 (1.00, 1.24)
E wave velocity		0.80 (0.74, 0.91)	1.27 (1.17, 1.59)
TR velocity		2.43 (2.30, 2.56)	2.86 (2.61, 3.17)
Fractional shortening		36.88 (28.57, 41.93)	45.89 (41.01, 51.57)
Medications	ACEi	3% (1)	40% (12)
	Loop diuretics	0% (0)	73% (22)
	Pimobendan	0% (0)	70% (21)
	Spironolactone	0% (0)	13% (4)
Weight (kg)		10.0 (7.4, 11.7)	8.9 (6.9, 11.3)

*Legend*: Data from patients with myxomatous mitral valve disease (n = 30) were included from their first visit in stages B1 and C. Categorical variables are reported as the proportion (frequency). Continuous variables are reported as the median (lower quartile, upper quartile). ACEi, angiotensin converting enzyme inhibitor; LA:Ao, left atrial to aortic root ratio; E wave velocity, peak transmitral flow in early diastole; LVIDDN, left ventricular internal diameter in diastole normalised to bodyweight (kg^0.294^); LVIDSN, left ventricular internal diameter in systole normalised to bodyweight (kg^0.315)^; TR, tricuspid regurgitation

### Association of molecular features with disease severity & analysis of paired samples belonging to unaffected controls

UPLC-MS detected a total of 6002 features from the extracted serum samples; 3737 in positive mode polarity (lipid RPC+) and 2265 in negative mode polarity (lipid RPC-). In the affected population, the intensity of 169 features (97 lipid RPC+, 72 lipid RPC-) were found to change significantly with progression to a more advanced stage of heart disease (Supplementary Table 2). In addition, 10 features (5 lipid RPC+, 5 lipid RPC-) differed between CKCS and non CKCS (Supplementary Table 3). Only one feature was associated with the interaction term between disease stage and breed (retention time: 3.4 min, mass-to-charge ratio: 915.53, polarity: RPC-, β: -1.32, Q: < 0.001), indicating that, for this feature, the relationship between its intensity and disease stage was different in each of the two breed groups.

For the lipid RPC + data there was a high degree of agreement between significant features observed in both OPLS-DA and mixed-effects models (Supplementary Fig. 1). Features that had markedly high/ low loadings were shown to have low Q-values in mixed-effects modelling. The OPLS-DA model for the lipid RPC- data was not significant, perhaps indicating the increased influence of individual variation in the species captured by this method.

Mixed-effects analysis found no significant differences in the serum lipidomes of healthy control animals at similarly spaced time-points.

### Annotation of metabolites that vary with disease stage

**Fig. 1:**
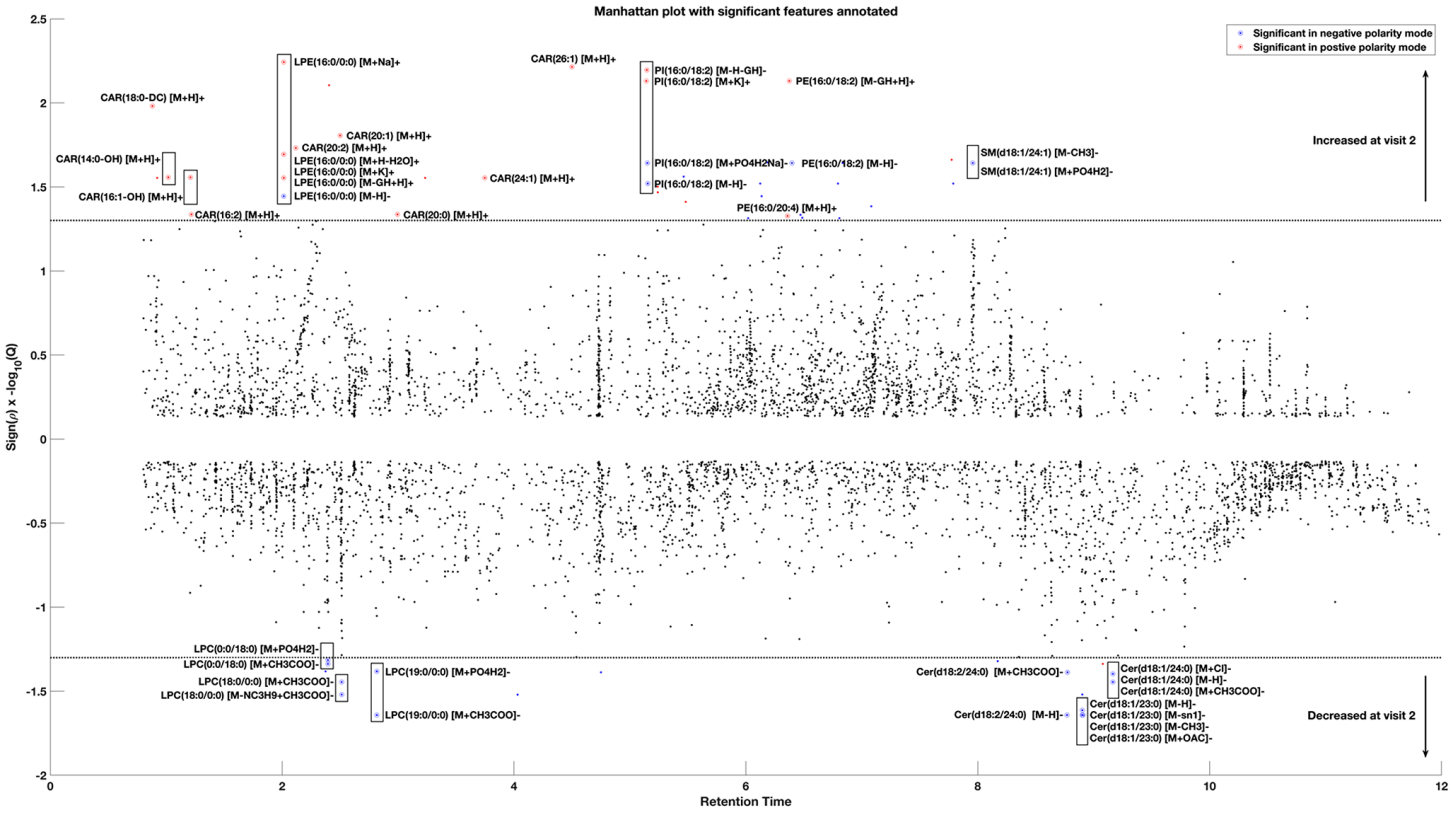
A Manhattan plot demonstrating the strength and direction of change in the intensity of 6002 features detected by ultra-performance liquid chromatography mass spectrometry across both positive and negative polarities. Legend: Results are displayed for ultra-performance liquid chromatography mass spectrometry (UPLC-MS) features identified in paired serum samples from 30 dogs with progressive myxomatous mitral valve disease. Using mixed-effects models 69 features showed a change that was significantly associated with development of heart failure and are coloured red (positive ionisation mode) or blue (negative ionisation mode), with the significance of results and direction of association indicated on the y axis. *Q* represents the false discovery rate adjusted value of the linear mixed effects model estimate and sign(ρ) gives the direction of change. The dotted line indicates a threshold where *Q* < 0.05. Of the features significantly associated with disease progression, features that were successfully annotated are labelled by molecule and ion type. CAR, acylcarnitine; Cer, ceramide; LPC, lysophosphatidylcholine; LPE, lysophosphatidylethanolamine; RPC+, reversed phase chromatography in positive ionisation mode; RPC-, reversed phase chromatography in negative ionisation mode; PE, phosphatidylethanolamine; PI, phosphoinositol; SM, sphingomyelin

Of the features that demonstrated significant differences in intensity between disease stage and/or breed groups (Q < 0.05), thirty-eight features were successfully annotated, yielding lipid identifications for 20 unique lipid species (Table 3). The precise location of double bonds or hydroxyl groups were not fully determinable, limiting the structural specificity of 12 lipid species. Whilst this may have impacted the ability to precisely identify a molecule, it would not affect the lipid species classification. The strength and direction of change for these lipids has been presented relative to the other 6002 UPLC-MS features in a Manhattan plot (Fig. 1). The small number of features associated with patient breed were not successfully annotated at this time. The mass to charge ratio (*m/z*) and retention time of these features are described in Supplementary Table 3.

**Table 3:** Annotated features that were significantly associated with progression from stage B1 to stage C (heart failure)

Annotation	Formula	Direction of Change	Ion Type	Polarity	β	*Q*
*CAR(14:0-OH)*	C21H41NO5	↑	[M + H]+	RPC+	0.78	0.028
*CAR(16:1-OH)*	C23H43NO5	↑	[M + H]+	RPC+	0.88	0.028
*CAR(16:2)*	C23H41NO4	↑	[M + H]+	RPC+	0.93	0.046
CAR(18:0-DC)	C25H47NO6	↑	[M + H]+	RPC+	0.95	0.010
CAR(20:0)	C27H53NO4	↑	[M + H]+	RPC+	0.83	0.046
*CAR(20:1)*	C27H51NO4	↑	[M + H]+	RPC+	1.11	0.016
*CAR(20:2)*	C27H49NO4	↑	[M + H]+	RPC+	0.98	0.019
CAR(24:1)	C31H59NO4	↑	[M + H]+	RPC+	0.92	0.028
CAR(26:1)	C33H63NO4	↑	[M + H]+	RPC+	0.94	0.006
Cer(d18:1/23:0)	C41H81NO3	↓	[M-CH3]-	RPC-	-0.81	0.023
			[M-H]-	RPC-	-0.82	0.024
			[M-sn1]-	RPC-	-0.81	0.023
			[M + OAC]-	RPC-	-0.82	0.023
Cer(d18:1/24:0)	C42H83NO3	↓	[M-H]-	RPC-	-0.82	0.036
			[M + CH3COO]-	RPC-	-0.81	0.036
			[M + Cl]-	RPC-	-0.80	0.040
Cer(d18:2/24:0)	C42H81NO3	↓	[M-H]-	RPC-	-0.72	0.023
			[M + CH3COO]-	RPC-	-0.64	0.041
LPC(0:0/18:0)	C26H54NO7P	↓	[M + CH3COO]-	RPC-	-0.58	0.046
			[M + PO4H2]-	RPC-	-0.54	0.048
LPC(18:0/0:0)	C26H54NO7P	↓	[M-NC3H9 + CH3COO]-	RPC-	-0.56	0.030
			[M + CH3COO]-	RPC-	-0.57	0.036
LPC(19:0/0:0)	C27H56NO7P	↓	[M + CH3COO]-	RPC-	-0.92	0.023
			[M + PO4H2]-	RPC-	-0.78	0.041
LPE(16:0/0:0)	C21H44NO7P	↑	[M-GH + H]+	RPC+	0.88	0.028
			[M-H]-	RPC-	0.70	0.036
			[M + H-H2O]+	RPC+	0.91	0.020
			[M + K]+	RPC+	0.91	0.020
			[M + Na]+	RPC+	1.24	0.006
*PE(16:0/18:2)*	C39H74NO8P	↑	[M-GH + H]+	RPC+	0.71	0.007
			[M-H]-	RPC-	0.63	0.023
*PE(16:0/20:4)*	C41H74NO8P	↑	[M + H]+	RPC+	0.74	0.047
*PI(16:0/18:2)*	C43H79O13P	↑	[M-H-GH]-	RPC+	1.00	0.007
			[M-H]-	RPC-	0.76	0.030
			[M + K]+	RPC+	0.93	0.007
			[M + PO4H2Na]-	RPC-	0.81	0.023
*SM(d18:1/24:1)*	C47H93N2O6P	↑	[M-CH3]-	RPC-	0.83	0.023
			[M + PO4H2]-	RPC-	0.85	0.023

Legend: Results are displayed for ultra-performance liquid chromatography mass spectrometry (UPLC-MS) features that displayed a significant association with disease progression (stage B1 to stage C) in dogs with myxomatous mitral valve disease. β is the coefficient of a linear mixed effects model that controlled for an effect of breed. Features were standardised and scaled prior to analysis. Q is the false discovery rate adjusted *P* value. Features where the annotated name is italicised contained a double bond or hydroxyl group whose structural location could not be ascertained. Upwards or downwards facing arrows are used to indicate if a lipid’s concentration increased or decreased in heart failure. CAR, acylcarnitine; Cer, ceramide; LPC, lysophosphatidylcholine; LPE, lysophosphatidylethanolamine; lipid RPC+, reversed phase chromatography in positive ionisation mode; lipid RPC-, reversed phase chromatography in negative ionisation mode; PE, phosphatidylethanolamine; PI, phosphoinositol; SM, sphingomyelin

**Fig. 2:**
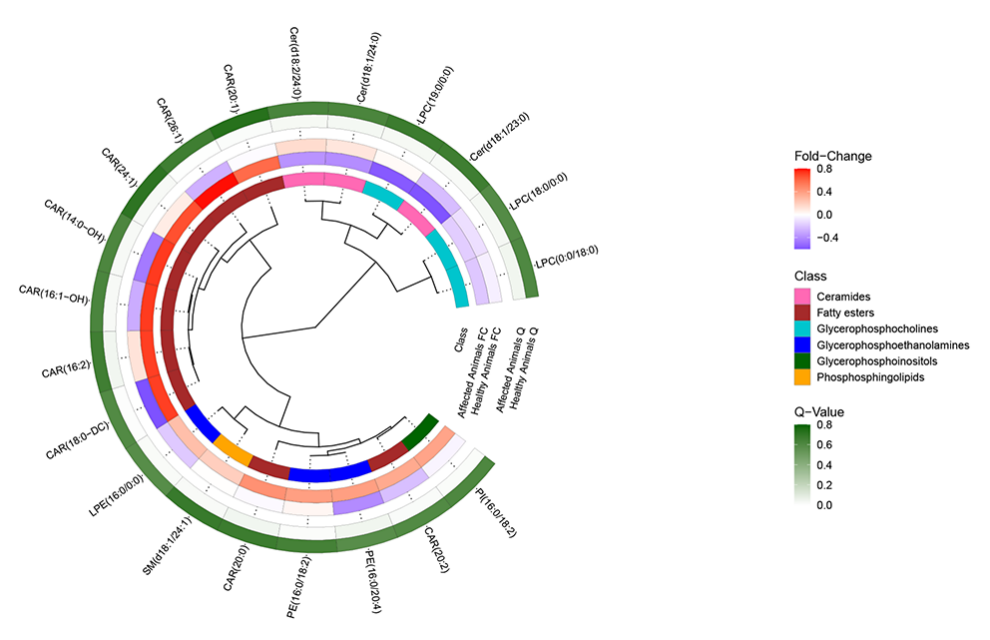
A heatmap showing the magnitude and direction of differences in feature intensity between visits for the control and affected populations. Legend: Figure 2 shows results for successfully annotated lipids displaying a significant association with disease severity. The dendrogram highlights how concentrations of these lipids differed in paired serum samples from dogs with myxomatous mitral valve disease that had progressed from stage B1 to C (heart failure). Results from a matched control group are provided as a point of comparison. Lipid-lipid associations (based on log_2_ fold-change) are displayed in a circular dendrogram (inner circle), and surrounded by heatmaps for lipid class, log_2_ fold-change in intensity between disease stage (affected animals) or visit (control animals) and significance as represented by the false discovery rate adjusted value of the mixed-effects model estimate (Q*)*. For clarity, where multiple ions were observed for a single lipid species the average values have been presented. Note, there were no significant differences between visits in samples from the control group. CAR, acylcarnitine; Cer, ceramide; LPC, lysophosphatidylcholine; LPE, lysophosphatidylethanolamine; PE, phosphatidylethanolamine; PI, phosphoinositol; SM, sphingomyelin

When interpreted in combination with the output of linear mixed effect models, individual lipid species were found to behave similarly to others within the same lipid class (Figs. 1 and 2). Circulating concentrations of lipids belonging to the following classes significantly increased in dogs after they had developed HF: acylcarnitines (CAR), lysophosphatidylethanolamines (LPE), phosphatidylethanolamines (PE), phosphoinositols (PI) and sphingomyelins (SM). In contrast, levels of lysophosphatidylcholine (LPC) and ceramide (Cer) species were significantly lower in dogs in HF, compared to their previous visit in stage B1. Further detail on the annotated lipids and their association with heart disease severity can be found in Table 3. When the change in these lipids was plotted for the control and affected populations (Fig. 2), the strength and, in some cases, direction of change was dissimilar for the two groups of dogs.

## Discussion

This untargeted metabolomic analysis demonstrated that the canine serum lipidome changes as dogs progress from preclinical disease to HF. Similar alterations did not occur in a control group of healthy dogs as they aged. This study is one of the first to analyse longitudinal data from dogs with this phenotype, providing robust results through the comparison of features at two time points in an accurately characterised cohort of patients. Regression models identified 169 UPLC-MS features that were differentially expressed in HF and, from these, 20 lipid species belonging to 7 lipid classes were successfully annotated. The results suggest that metabolic alterations occur within individuals and this may be due to a shift in substrate use with advancing MMVD. The finding that most robustly supports this claim is the increased concentration of circulating acylcarnitines in HF. A larger, prospective cohort is needed to confirm these preliminary results.

**Fig. 3:**
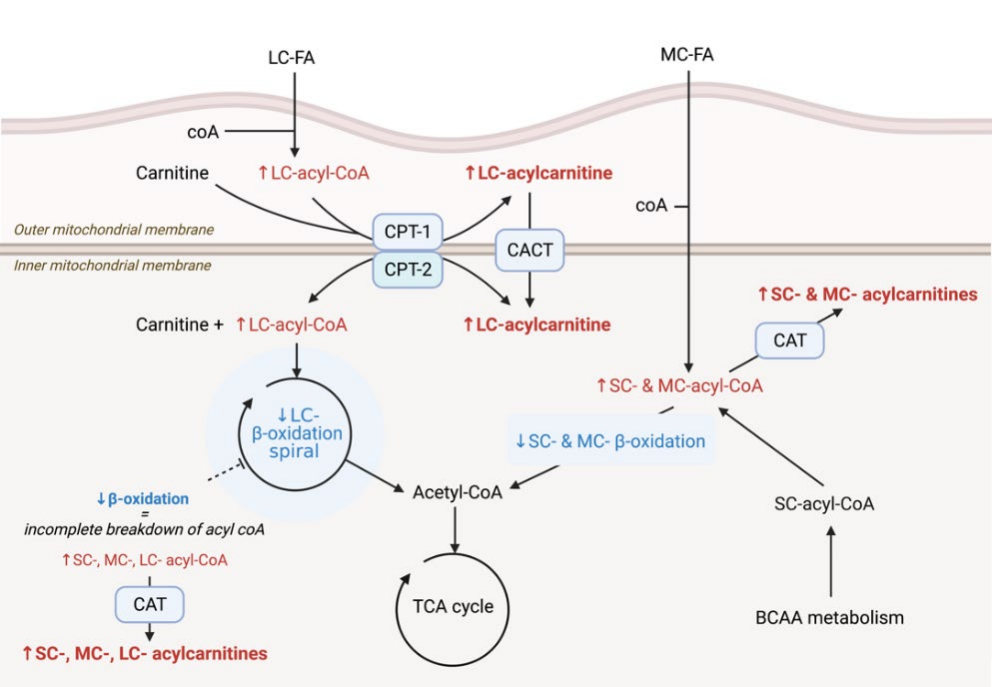
Methods by which acylcarnitine concentrations could increase secondary to reduced β-oxidation. Acylcarnitines are thought to increase as a result of reduced or impaired catabolism of acyl-CoAs. Additionally, if pathways involved in transporting fatty acids (FA) into mitochondria are affected, acylcarnitine formation in the cytoplasm may protect against acyl-CoA accumulation. The export of acylcarnitines into the circulation is poorly described. BCAA, branched chain amino acids; CACT, carnitine acylcarnitine translocase; CAT, carnitine acyltransferase; CPT, carnitine palmitoyltransferase; SC, short chain; MC, medium chain; LC, long chain; TCA, tricarboxylic acid cycle

Our finding that concentrations of some long chain (LC) acylcarnitines were increased in HF, concurs with the published literature. In human patients, acylcarnitine concentrations have been consistently associated with adverse clinical outcomes (Ahmad et al., 2016; Guasch-Ferré et al., 2016; Kalim et al., 2013; Rizza et al., 2014; Shah et al., 2010; Strand et al., 2017), and values correlate with NT-proBNP, a marker of disease severity (Ruiz et al., 2017). In previous canine studies, concentrations were greater in MMVD compared to healthy controls (Li et al., 2015) and consistent with the present study, this difference is more pronounced once patients have developed HF (Li et al., 2021). The present study is the first to observe this within client-owned patients as their disease progressed. If β-oxidation is impaired, fatty acyl-coAs accumulate in cytosolic and mitochondrial pools, subsequently increasing the production and cellular efflux of acylcarnitines. (Makrecka-Kuka et al., 2017; McCoin et al., 2015). Examining the structure of perturbed lipids can provide further information on the mechanism by which alterations in metabolism occur (Fig. 3) (Makrecka-Kuka et al., 2017; Ruiz et al., 2017). In this study, lipids with a chain length ≥ 22 carbon atoms, or containing a hydroxylated (OH-) or dicarboxylic (DC-) group were increased in HF alongside LC-acylcarnitines. Given that these lipids are related to metabolism in peroxisomes and the endoplasmic reticulum (Rizzo et al., 2003; Ruiz et al., 2017; Wanders & Waterham, 2006), our results suggest that dysregulation of FA metabolism may extend beyond the mitochondria in dogs with MMVD. This concurs with previous findings in human patients and, interestingly, peroxisomal metabolism is regulated by some of the same transcription factors that are thought to alter mitochondrial metabolism in cardiac disease (peroxisome proliferator activated receptors) (Ruiz et al., 2017). Together, these results show that the way acylcarnitine is processed differed between stages of MMVD. As acylcarnitine concentrations mirror the amount of acyl-CoA within the mitochondria, this most likely reflects a reduction in β-oxidation in HF, either within or remote to the myocardium (ter Veld et al., 2009). Compared to the other lipid species identified in this study, an increase in long chain, very long chain, OH- and DC- acylcarnitine concentrations presents the most convincing evidence of disordered substrate use in advanced MMVD.

Our findings build upon previous work that has been conducted in MMVD using metabolomic and transcriptomic approaches. Evidence of altered fatty acid and glucose metabolism has been observed in dogs with MMVD when serum and cardiac tissue from affected individuals are compared to healthy controls. Notably, by comparing serum samples from two points in the course of disease, our study adds to these results by suggesting that changes are associated with disease progression within individual dogs (Li et al., 2015). This finding is consistent with previous analyses of the products of lipid metabolism in humans, where reduced rates of β-oxidation correlate with disease severity (De las Fuentes et al., 2003; Otsuka et al., 2002). In dogs, decreased FA metabolism was seen as a late stage phenomenon in experimental models of ischaemic and pacing induced HF (Chandler et al., 2004; Recchia et al., 1998). Dogs with naturally occurring MMVD may therefore develop alterations in myocardial metabolism as a maladaptive response to increasing disease severity. These changes may play a causal role in furthering disease progression and, of particular relevance to MMVD, it is hypothesised that deranged myocardial metabolism contributes to the development of pathological hypertrophy (Gibb & Hill, 2018; Nakamura & Sadoshima, 2018). As this was an analysis of serum, it should be noted that direct conclusions regarding myocardial metabolism cannot be drawn, however through comparison to other studies, including those in MMVD (Li et al., 2015), it is possible to speculate on their relevance in the pathophysiology of this disease.

### General Strengths and Limitations

A particular strength of this analysis was the phenotype studied. In humans, the development of cardiovascular disease is compounded by chronic exposure to physiological and environmental risk factors (Kannel & McGee, 1979). The components of the metabolome dynamically change in response to these conditions, which can confound studies investigating associations with HF severity. To this end, several of the lipid species identified in this study have been linked to established dietary and lifestyle risk factors (Goldenberg et al., 2019; Guasch-Ferré et al., 2016; Lahey et al., 2014). As dogs have a more stable diet and lifestyle than human patients and MMVD is believed to have a genetic basis, confounding effects would be reduced (Lewis et al., 2011; Lloyd et al., 2017; Olsen et al., 1999; Swenson et al., 1996). The study also benefitted from selecting dogs from a separate longitudinal cohort study, in which over 400 dogs had been assessed over several years using accurate and objective methods. Residual blood samples had been consistently handled according to a standardised protocol and metadata surrounding the events of their acquisition were available. Altogether, this meant that relevant criteria could be applied based on prior research, to select a well phenotyped population in a way that would be difficult to reproduce in human patients.

Despite this, potential confounders still exist within the data. This was a retrospective analysis of client owned dogs, meaning that clinical and environmental factors were not controlled. Specifically, dogs were not on a standardised diet or therapeutic protocol and for many dogs these factors differed between time points (Lloyd et al., 2017); Supplementary Tables 4 and 5 contain information that had been recorded about diet and medications. Blood sampling was conducted at different times of day and the duration of time between feeding and sampling was not consistent. Non-cardiac comorbidities were present and their prevalence increased at the second examination (Supplementary Table 5). Notably, one dog in the affected population had developed diabetes mellitus (DM) when examined in HF which was medically controlled. Unlike human cardiovascular disease, DM is not a risk factor for MMVD, however it should be noted that DM is expected to affect metabolism (Lopaschuk et al., 2010). This is unlikely to have biased results but may have introduced noise into the data used for analysis. Though multivariable methods were applied, the number of explanatory variables was limited by a lack of statistical power. Instead, using paired samples allowed each patient to act as its own control. As these data were captured from two visits a number of years apart, it is possible that some changes occurred due to aging or sample stability in storage (Houtkooper et al., 2011; Strand et al., 2017). To evaluate this, the study included an analysis of a separate control population that had been matched to the affected cohort. There were no significant differences in the lipidome between visits for these dogs and, when visually assessed, the species related to disease severity showed dissimilar patterns of change in the control group (Fig. 2). The age at second examination differed between the two groups, with control dogs being younger than cases on average. With age, FA oxidation decreases in both myocardial and skeletal muscle and we cannot conclusively exclude this possibility (Toth & Tchernof, 2000). Having acknowledged these limitations, this also represent a study strength, as studies conducted in a natural setting produce results that are more generalisable that can be applied to the naturally occurring phenotype.

As serum samples were used for analysis, our results cannot be used to infer that changes occurred in myocardial metabolism directly. Metabolic derangement in HF is understood to extend beyond the myocardium (Rosano & Vitale, 2018), therefore these results may reflect secondary effects of HF on other systems, or changes that occur as part of a syndrome of heart failure. In the present study, one such example is the reduction in weight observed in dogs that developed HF, which could be attributed to changes in appetite or cachexia (Boswood et al., 2016; Ineson et al., 2019). Other studies have observed correlations between the products of myocardial metabolism in tissue samples and the circulation, so it is possible that there is some crossover (Makrecka-Kuka et al., 2017). A parallel analysis of myocardial tissue would be required to test this. Interpretation is also limited by the fact that relevant pathways are complex and poorly understood and existing research on this topic focusses on human cardiovascular diseases. Though arteriosclerosis and inflammation are described in canine MMVD, they are present to a lesser degree which may account for some of the discrepancies when findings are compared to studies of human patients (Falk et al., 2010). This point may be of relevance for the sphingolipids (Cer(d18:1/23:0), Cer(d18:1/24:0), Cer(d18:2/24:0), SM(d18:0/24:1)) that were differentially expressed when dogs were in HF. Ceramides and sphingomyelins have been primarily studied in ischaemic heart disease and increased concentrations of some ceramides are consistently recognised as risk factors. In contrast, in this study, C24:0 and C23:0 were present at lower concentrations in dogs with HF. C24:0 has been both positively, (Anroedh et al., 2018; Ji et al., 2017; Wang et al., 2017) and negatively associated with risk (Laaksonen et al., 2016; Peterson et al., 2018; Tarasov et al., 2014), whilst C23:0 is poorly described in the published literature. Further research is required to validate and understand the mechanisms behind changes that occur in canine MMVD.

Finally, this study was an analysis of the products of lipid metabolism and interpretation is limited to the molecules that could be annotated. Whilst a difference in the lipidome was observed in HF, this study did not examine whether simultaneous changes in glucose, ketones or amino acids also occur in MMVD.

## Conclusions

This study presents evidence that the lipid metabolome of dogs is altered in advanced MMVD. By comparing paired samples from patients in stages B1 and C, serum concentrations of LC-acylcarnitines, sphingolipids and glycerophospholipids were found to change with disease progression, suggesting that metabolism alters with the development of HF. Differences in the concentrations of some of these species, particularly acylcarnitines, is consistent with accumulation of fatty acyl-CoAs secondary to reduced β-oxidation. However, substrate selection is not responsible for all observed results. Because of the prolonged preclinical phase of disease, dogs with MMVD could receive interventions targeting myocardial metabolism as a mechanism to delay disease progression. Further research is needed before this hypothesis can be explored.

## Electronic supplementary material

Below is the link to the electronic supplementary material.

Supplementary Material 1

## Data Availability

The metabolomics and metadata reported in this paper are available via Metabolights (https://www.ebi.ac.uk/metabolights/index) study identifier MTBLS716.

## List of Abbreviations

ACEi: Angiotensin converting enzyme inhibitor
ACVIM: American College of Veterinary Internal Medicine
ATP: Adenosine triphosphate
CAR: Acylcarnitine
Cer: Ceramide
CKCS: Cavalier King Charles Spaniel
E wave velocity: Peak transmitral flow in early diastole
FA: Fatty acid
HF: Heart failure
LA:Ao: Left atrial to aortic root ratio
Lipid RPC+: UPLC-MS for lipids, positive polarity ionization
Lipid RPC-: UPLC-MS for lipids, negative polarity ionization
LPC: Lysophosphatidylcholine
LPE: Lysophosphatidylethanolamine
LVIDDN: Normalised left ventricular internal diameter at end diastole
LVISDN: Normalised left ventricular internal diameter at end systole
MMVD: Myxomatous mitral valve disease
*m/z*: Mass to charge ratio
OPLS-DA: Orthogonal projection to latent structure discriminant analysis
PE: Phosphatidylethanolamine
PI: Phosphoinositol
SM: Sphingomyelin
TR: Tricuspid regurgitation
UPLC-MS: Ultra-performance liquid chromatography mass spectrometry
