# Supplementary material for: Evidence of altered fatty acid metabolism in dogs with naturally occurring valvular heart disease and congestive heart failure
Source: Metabolomics. 2022 May 30;18(6):34. doi: 10.1007/s11306-022-01887-7 (PMC9151558; doi:10.1007/s11306-022-01887-7)
Supplement: Supplementary file 1 — Supplementary Material 1 [file 11306_2022_1887_MOESM1_ESM.docx]

**Supplementary Materials**

**Supplementary Methods.**

Each sample analysis was performed in a random order designed to show no correlation or other relationship to study design and outcome. To facilitate quality assessment and pre-processing, a pooled quality control sample was prepared by combining equal parts of each study sample and analysed at 10 study sample intervals during sample analyses. An additional set of quality control sample dilutions was created (10x 100%, 5x 80%, 3x 60%, 3x 40%, 5x 20%, 10x 1%) and analysed at the start and end of each set of sample analyses for assessment of analyte response.

Serum samples were prepared and analysed as previously described (Izzi-Engbeaya et al. 2018; Lewis et al. 2016). To prepare serum samples, 50 μL aliquots were taken from each sample and the pooled quality control and diluted 1:1 volume/volume (v/v) with ultrapure water. Protein was removed by addition of organic solvent (diluted sample/isopropanol in 1:4 v/v ratio). Mixtures of method-specific authentic chemical standards were added at the protein precipitation step to monitor data quality during acquisition. The sample mixture was incubated whilst shaking for 2 hours at 4°C and subsequently centrifuged (3486 x g for 10 minutes at 4°C) to separate precipitated protein from homogenous supernatant. This was decanted and used for ultra-performance liquid chromatography mass spectrometry (UPLC-MS) analysis. Serum analyses were performed on ACQUITY UPLC instruments (Waters Corp., Milford, MA, USA) coupled to Xevo G2‐S Q-TOF mass spectrometers (Waters Corp., Manchester, UK) via a Z‐spray electrospray ionisation (ESI) source operating in both positive and negative ionisation modes to generate lipid positive (lipid RPC+) and negative (lipid RPC-) datasets.

Profiling was performed using a 2.1×100 mm BEH C8 column, thermostated at 55°C. Prepared samples (2 uL for negative mode and 1 uL for positive mode) were injected into the chromatographic system using full loop mode. Solvent A consisted of water:isopropanol:acetonitrile 2:1:1, 5 mM ammonium acetate, 0.05% acetic acid, 20 µM phosphoric acid. Solvent B consisted of isopropanol:acetonitrile 1:1, 5 mM ammonium acetate, 0.05% acetic acid. The chromatographic gradient program is provided in Supplementary Table 1. The mass spectrometers were set up as follows: capillary voltage 1.5 kV for negative mode and 2 kV for positive mode; sample cone voltage 25 V; desolvation gas flow 1000L.h-1; cone gas flow 150L.h-1; source temperature 120°C; desolvation temperature 600°C. A scan range of 50-2000m.z-1 in centroid mode was used to collect the data. Leucine enkephalin was used for Lockspray mass correction.

Following raw data acquisition, peak alignment, peak integration, and deconvolution (isotope grouping) tasks were performed to extract chemical measurements (features) from the raw data using Progenesis QI (Waters Corporation). All datasets were pre-processed using the nPYc-Toolbox (Sands et al. 2019), including elimination of potential run-order effects and feature filtering. Only features measured with high analytical quality (relative standard deviation in pooled quality control < 20%, pooled quality control dilution series Pearson correlation to dilution factor > 0.9, relative standard deviation in study samples > 1.1* relative standard deviation in pooled quality control) were retained and put forward for further statistical analysis. After feature filtering, datasets contained the following number of variables; positive polarity ionization (lipid RPC+): 3737; negative polarity ionization (lipid RPC-): 2265.

**Supplementary Table 1.** Gradient conditions for reverse phase lipid separation

| Step | Time (mins) | Flow (mL.min-1) | % A |
| --- | --- | --- | --- |
| 1 | Initial | 0.6 | 99.0 |
| 2 | 0.10 | 0.6 | 99.0 |
| 3 | 2.00 | 0.6 | 70.0 |
| 4 | 11.50 | 0.6 | 10.0 |
| 5 | 12.00 | 1.0 | 0.1 |
| 6 | 12.5 | 1.0 | 0.1 |
| 7 | 12.55 | 0.9 | 35.0 |
| 8 | 12.65 | 0.8 | 70.0 |
| 9 | 12.75 | 0.7 | 99.0 |
| 10 | 12.95 | 0.6 | 99.0 |
| 11 (sample load) | 15.00 | 0.6 | 99.0 |

**Supplementary Table 2.** The results of mixed-effects models analysing the association between disease stage and feature intensity in positive and negative ionisation modes.

| Retention Time | m/z | β | Q | Polarity |
| --- | --- | --- | --- | --- |
| 0.800 | 225.198 | 0.774 | 0.034 | RPC+ |
| 0.830 | 111.007 | 0.875 | 0.048 | RPC- |
| 0.850 | 442.352 | 0.959 | 0.007 | RPC+ |
| 0.880 | 411.287 | 1.086 | 0.006 | RPC+ |
| 0.880 | 458.348 | 0.949 | 0.010 | RPC+ |
| 0.880 | 482.342 | 0.834 | 0.017 | RPC+ |
| 0.920 | 382.295 | 0.531 | 0.028 | RPC+ |
| 0.930 | 466.329 | 0.632 | 0.020 | RPC+ |
| 1.020 | 388.306 | 0.783 | 0.028 | RPC+ |
| 1.060 | 484.363 | 0.742 | 0.009 | RPC+ |
| 1.070 | 472.363 | 0.727 | 0.025 | RPC+ |
| 1.080 | 394.295 | 0.937 | 0.008 | RPC+ |
| 1.180 | 438.321 | 0.970 | 0.006 | RPC+ |
| 1.210 | 414.322 | 0.882 | 0.028 | RPC+ |
| 1.220 | 396.311 | 0.926 | 0.046 | RPC+ |
| 1.250 | 227.116 | 1.090 | 0.005 | RPC+ |
| 1.290 | 486.379 | 0.649 | 0.010 | RPC+ |
| 1.310 | 420.310 | 0.778 | 0.015 | RPC+ |
| 1.320 | 440.336 | 0.856 | 0.036 | RPC+ |
| 1.320 | 595.288 | 0.655 | 0.041 | RPC- |
| 1.330 | 372.311 | 0.835 | 0.020 | RPC+ |
| 1.460 | 416.337 | 0.967 | 0.010 | RPC+ |
| 1.570 | 386.326 | 0.565 | 0.034 | RPC+ |
| 1.610 | 442.353 | 0.879 | 0.026 | RPC+ |
| 1.650 | 448.342 | 0.867 | 0.009 | RPC+ |
| 1.830 | 400.343 | 0.738 | 0.019 | RPC+ |
| 1.830 | 450.357 | 1.128 | 0.001 | RPC+ |
| 1.900 | 313.274 | 0.898 | 0.016 | RPC+ |
| 1.900 | 492.249 | 0.926 | 0.015 | RPC+ |
| 1.940 | 617.369 | 0.610 | 0.023 | RPC- |
| 10.200 | 259.241 | 0.751 | 0.034 | RPC+ |
| 10.200 | 287.273 | 0.747 | 0.028 | RPC+ |
| 10.210 | 219.209 | 0.830 | 0.017 | RPC+ |
| 2.010 | 313.274 | 0.877 | 0.028 | RPC+ |
| 2.010 | 436.283 | 0.908 | 0.020 | RPC+ |
| 2.010 | 452.277 | 0.704 | 0.036 | RPC- |
| 2.010 | 454.294 | 0.912 | 0.019 | RPC+ |
| 2.010 | 476.276 | 1.240 | 0.006 | RPC+ |
| 2.010 | 492.249 | 0.912 | 0.020 | RPC+ |
| 2.050 | 476.373 | 0.937 | 0.006 | RPC+ |
| 2.120 | 452.372 | 0.979 | 0.019 | RPC+ |
| 2.130 | 470.380 | 0.884 | 0.017 | RPC+ |
| 2.140 | 325.272 | 1.099 | 0.030 | RPC- |
| 2.170 | 754.523 | 0.768 | 0.033 | RPC+ |
| 2.180 | 710.501 | 0.837 | 0.015 | RPC+ |
| 2.200 | 452.365 | 0.975 | 0.012 | RPC+ |
| 2.270 | 761.005 | 0.732 | 0.049 | RPC+ |
| 2.290 | 707.964 | 0.733 | 0.042 | RPC+ |
| 2.370 | 659.350 | -0.849 | 0.041 | RPC- |
| 2.390 | 582.377 | -0.575 | 0.046 | RPC- |
| 2.390 | 620.334 | -0.538 | 0.048 | RPC- |
| 2.390 | 756.264 | -0.755 | 0.023 | RPC- |
| 2.390 | 974.194 | -0.729 | 0.036 | RPC- |
| 2.400 | 133.100 | 0.869 | 0.009 | RPC+ |
| 2.400 | 145.101 | 0.945 | 0.006 | RPC+ |
| 2.400 | 147.116 | 0.977 | 0.007 | RPC+ |
| 2.400 | 149.131 | 0.940 | 0.016 | RPC+ |
| 2.400 | 163.147 | 1.021 | 0.006 | RPC+ |
| 2.400 | 187.147 | 0.917 | 0.014 | RPC+ |
| 2.400 | 189.163 | 0.995 | 0.006 | RPC+ |
| 2.400 | 201.163 | 0.957 | 0.006 | RPC+ |
| 2.400 | 213.163 | 0.913 | 0.008 | RPC+ |
| 2.400 | 269.227 | 0.978 | 0.006 | RPC+ |
| 2.400 | 269.308 | 0.911 | 0.026 | RPC+ |
| 2.400 | 269.389 | 0.955 | 0.016 | RPC+ |
| 2.400 | 81.070 | 1.086 | 0.006 | RPC+ |
| 2.400 | 95.086 | 0.992 | 0.006 | RPC+ |
| 2.500 | 454.389 | 1.114 | 0.016 | RPC+ |
| 2.510 | 523.303 | -0.562 | 0.030 | RPC- |
| 2.510 | 582.377 | -0.569 | 0.036 | RPC- |
| 2.510 | 664.379 | -0.781 | 0.024 | RPC- |
| 2.720 | 643.363 | 0.555 | 0.034 | RPC+ |
| 2.780 | 372.291 | 0.918 | 0.016 | RPC+ |
| 2.820 | 596.392 | -0.915 | 0.023 | RPC- |
| 2.820 | 634.349 | -0.780 | 0.041 | RPC- |
| 2.990 | 456.405 | 0.831 | 0.046 | RPC+ |
| 3.230 | 212.198 | 1.095 | 0.026 | RPC+ |
| 3.230 | 293.284 | 1.074 | 0.028 | RPC+ |
| 3.230 | 310.304 | 1.073 | 0.028 | RPC+ |
| 3.350 | 293.284 | 1.104 | 0.019 | RPC+ |
| 3.750 | 510.452 | 0.921 | 0.028 | RPC+ |
| 3.780 | 764.547 | 1.101 | 0.006 | RPC+ |
| 3.930 | 536.472 | 0.974 | 0.006 | RPC+ |
| 4.020 | 791.414 | -0.866 | 0.018 | RPC+ |
| 4.030 | 751.443 | -0.809 | 0.030 | RPC- |
| 4.130 | 766.478 | -0.538 | 0.048 | RPC- |
| 4.270 | 793.430 | -0.773 | 0.036 | RPC+ |
| 4.370 | 905.524 | -0.765 | 0.024 | RPC- |
| 4.500 | 538.483 | 0.937 | 0.006 | RPC+ |
| 4.640 | 916.587 | 0.824 | 0.034 | RPC+ |
| 4.750 | 351.325 | -0.834 | 0.041 | RPC- |
| 4.820 | 330.060 | 0.782 | 0.039 | RPC+ |
| 4.840 | 860.539 | -0.555 | 0.048 | RPC- |
| 5.000 | 557.456 | -0.786 | 0.032 | RPC- |
| 5.090 | 560.475 | -0.651 | 0.015 | RPC+ |
| 5.140 | 873.489 | 0.933 | 0.007 | RPC+ |
| 5.150 | 575.503 | 0.995 | 0.006 | RPC+ |
| 5.150 | 833.520 | 0.759 | 0.030 | RPC- |
| 5.150 | 953.478 | 0.809 | 0.023 | RPC- |
| 5.230 | 1002.593 | 0.826 | 0.023 | RPC- |
| 5.240 | 177.163 | 0.402 | 0.028 | RPC+ |
| 5.240 | 302.224 | 0.467 | 0.047 | RPC+ |
| 5.240 | 351.305 | 0.382 | 0.026 | RPC+ |
| 5.240 | 587.445 | 0.442 | 0.031 | RPC+ |
| 5.240 | 589.461 | 0.375 | 0.034 | RPC+ |
| 5.300 | 388.063 | 0.619 | 0.048 | RPC- |
| 5.320 | 564.532 | 0.902 | 0.006 | RPC+ |
| 5.360 | 899.509 | 0.876 | 0.007 | RPC+ |
| 5.460 | 984.595 | 0.662 | 0.027 | RPC- |
| 5.480 | 589.513 | 0.775 | 0.039 | RPC+ |
| 5.640 | 955.493 | 0.845 | 0.048 | RPC- |
| 5.650 | 577.519 | 0.788 | 0.015 | RPC+ |
| 5.820 | 603.535 | 0.651 | 0.033 | RPC+ |
| 6.020 | 1223.764 | 0.519 | 0.048 | RPC- |
| 6.120 | 581.183 | 0.662 | 0.030 | RPC- |
| 6.140 | 1012.626 | 0.508 | 0.036 | RPC- |
| 6.170 | 1207.769 | 0.840 | 0.023 | RPC- |
| 6.360 | 599.504 | 0.666 | 0.049 | RPC+ |
| 6.360 | 740.524 | 0.737 | 0.047 | RPC+ |
| 6.370 | 575.505 | 0.706 | 0.007 | RPC+ |
| 6.380 | 738.509 | 0.581 | 0.036 | RPC- |
| 6.400 | 714.509 | 0.626 | 0.023 | RPC- |
| 6.420 | 858.572 | 0.577 | 0.048 | RPC- |
| 6.470 | 1002.609 | 0.790 | 0.046 | RPC- |
| 6.490 | 1052.657 | 0.666 | 0.048 | RPC- |
| 6.490 | 682.635 | 0.696 | 0.017 | RPC+ |
| 6.650 | 1277.812 | 0.720 | 0.036 | RPC- |
| 6.750 | 818.589 | 0.908 | 0.023 | RPC- |
| 6.760 | 1042.842 | 0.688 | 0.037 | RPC- |
| 6.790 | 1066.673 | 0.710 | 0.030 | RPC- |
| 6.810 | 1040.657 | 0.542 | 0.048 | RPC- |
| 6.830 | 1261.818 | 0.791 | 0.023 | RPC- |
| 7.080 | 1054.672 | 0.555 | 0.041 | RPC- |
| 7.480 | 907.633 | 0.631 | 0.048 | RPC- |
| 7.560 | 738.620 | 0.734 | 0.049 | RPC+ |
| 7.770 | 664.625 | 0.677 | 0.036 | RPC+ |
| 7.770 | 882.647 | 0.715 | 0.022 | RPC+ |
| 7.790 | 940.650 | 0.704 | 0.030 | RPC- |
| 7.960 | 521.315 | 0.775 | 0.031 | RPC- |
| 7.960 | 581.301 | 0.799 | 0.023 | RPC- |
| 7.960 | 677.412 | 0.835 | 0.036 | RPC- |
| 7.960 | 695.418 | 0.842 | 0.023 | RPC- |
| 7.960 | 797.655 | 0.830 | 0.023 | RPC- |
| 7.960 | 871.687 | 0.858 | 0.023 | RPC- |
| 7.960 | 909.649 | 0.852 | 0.023 | RPC- |
| 7.970 | 611.318 | 0.810 | 0.039 | RPC- |
| 8.000 | 824.666 | 0.638 | 0.020 | RPC- |
| 8.000 | 922.637 | 0.552 | 0.023 | RPC- |
| 8.170 | 632.562 | -0.789 | 0.048 | RPC- |
| 8.200 | 678.605 | -0.621 | 0.037 | RPC- |
| 8.250 | 729.635 | 0.373 | 0.045 | RPC+ |
| 8.350 | 606.583 | -0.723 | 0.048 | RPC- |
| 8.770 | 646.614 | -0.723 | 0.023 | RPC- |
| 8.770 | 706.636 | -0.643 | 0.041 | RPC- |
| 8.790 | 682.625 | -0.970 | 0.023 | RPC- |
| 8.900 | 378.372 | -0.813 | 0.023 | RPC- |
| 8.900 | 586.592 | -0.773 | 0.036 | RPC- |
| 8.900 | 634.615 | -0.821 | 0.024 | RPC- |
| 8.900 | 680.621 | -0.794 | 0.030 | RPC- |
| 8.900 | 694.636 | -0.824 | 0.023 | RPC- |
| 8.910 | 670.591 | -0.813 | 0.023 | RPC- |
| 9.080 | 615.480 | -0.856 | 0.046 | RPC+ |
| 9.080 | 632.460 | -0.728 | 0.034 | RPC+ |
| 9.110 | 640.629 | -0.777 | 0.039 | RPC+ |
| 9.170 | 648.630 | -0.823 | 0.036 | RPC- |
| 9.170 | 684.607 | -0.803 | 0.040 | RPC- |
| 9.170 | 708.652 | -0.808 | 0.036 | RPC- |
| 9.280 | 674.649 | -0.596 | 0.023 | RPC- |
| 9.300 | 650.646 | -0.600 | 0.037 | RPC- |

*Legend:* The table displays the ultra-performance liquid chromatography mass spectrometry characteristics (mass to charge ratio and retention time) of features significantly associated with progression from stage B1 to stage C (heart failure) in dogs with myxomatous mitral valve disease. Results are provided for the 97 significant features found in positive polarity ionization (lipid RPC+) and 72 significant features found in negative polarity ionization (lipid RPC-). Results are reported as the coefficient (β) which represents the strength and direction of an association between two variables. Q represents the false discovery rate adjusted P-value. Features highlighted in blue were successfully annotated.

**Supplementary Table 3.** The results of mixed-effects models analysing the association between breed and feature intensity in positive and negative ion modes.

| Retention Time | m/z | β | *Q* | Polarity |
| --- | --- | --- | --- | --- |
| 0.800 | 222.036 | 1.549 | .025 | RPC+ |
| 0.810 | 338.892 | 1.563 | .025 | RPC+ |
| 3.400 | 915.532 | 2.093 | < .001 | RPC- |
| 3.800 | 782.498 | -1.370 | .049 | RPC- |
| 4.520 | 738.528 | -1.475 | .032 | RPC+ |
| 4.710 | 708.516 | -1.430 | .044 | RPC+ |
| 4.980 | 927.374 | 1.423 | .031 | RPC- |
| 5.280 | 883.817 | 1.583 | .006 | RPC- |
| 5.430 | 736.548 | -1.435 | .044 | RPC+ |
| 7.270 | 710.551 | -1.548 | .006 | RPC- |

*Legend:* The table displays the ultra-performance liquid chromatography mass spectrometry characteristics (mass to charge ratio and retention time) of features significantly associated with breed (Cavalier King Charles Spaniel (CKCS)/ non-CKCS) in dogs with myxomatous mitral valve disease. Results are provided for the 5 significant features found in positive polarity ionization (lipid RPC+) and 5 significant features found in negative polarity ionization (lipid RPC-). Results are reported as the coefficient (β) which represents the strength and direction of an association between two variables. Q represents the false discovery rate adjusted P-value. Retention time was measured in minutes. Features highlighted in blue were selected for further identification efforts.

**Supplementary Table 4.** Dietary intake of dogs in the affected and control populations.

| Group | Breed | Diet (Visit 1) | Diet (Visit 2) |
| --- | --- | --- | --- |
| MMVD | CKCS | Hills h/d + meat + fish | Hills h/d + meat + fish |
| MMVD | CKCS | Pedigree | Pedigree |
| MMVD | CKCS | Unspecified | Unspecified |
| MMVD | CKCS | Unspecified | Unspecified brand. Low fat prescription food |
| MMVD | CKCS | Hills r/d + chicken | Hills r/d + chicken |
| MMVD | CKCS | Unspecified | Chicken + rice + pasta |
| MMVD | CKCS | Hills d/d | Hills d/d |
| MMVD | CKCS | Sainsburys own brand | Caesar |
| MMVD | CKCS | Unspecified | Unspecified |
| MMVD | CKCS | Burns + chicken | Burns + chicken |
| MMVD | CKCS | Unspecified dry food + liver + chicken | Unspecified dry food + liver + chicken |
| MMVD | CKCS | Wainwrights | Lily’s Kitchen |
| MMVD | CKCS | Pedigree + cooked food | Cooked food |
| MMVD | CKCS | Unspecified obesity prescription diet + Caesar + chicken | Unspecified dry food + Caesar + chicken |
| MMVD | CKCS | Unspecified | Unspecified |
| MMVD | CKCS | Bakers | Sainsburys own brand |
| MMVD | CKCS | Hills r/d | Hills r/d |
| MMVD | CKCS | Unspecified | Unspecified |
| MMVD | CKCS | Unspecified | Cooked food |
| MMVD | CKCS | Natural Instinct | Natural Instinct |
| MMVD | Bichon Frisé | Unspecified | Unspecified |
| MMVD | Chihuahua | Raw meat + vegetables | Raw meat + vegetables |
| MMVD | Cocker Spaniel | Unspecified | Hills z/d |
| MMVD | Crossbreed | Unspecified | Chicken + rice |
| MMVD | Crossbreed | Hills Science Plan | Hills Science Plan |
| MMVD | Crossbreed | Arden Grange | Cooked food |
| MMVD | Maltese | Unspecified | Cooked food |
| MMVD | Shih Tzu | Unspecified | Unspecified dog food + meat |
| MMVD | Shih Tzu | Hills Science Plan | Hills i/d, Hills w/d + chicken |
| MMVD | Yorkshire Terrier | Meat + fish + rice | Meat + fish + rice |
| Control | CKCS | Bakers Weight Control | Unspecified |
| Control | CKCS | Burns | Lily’s Kitchen |
| Control | CKCS | Cooked food | Unspecified |
| Control | CKCS | Burns + meat | Unspecified |
| Control | CKCS | Cooked food | Unspecified dry food + cooked food |
| Control | Jack Russell Terrier | Pedigree | Bakers |
| Control | Poodle | Hills Science Plan | Hills Science Plan |
| Control | Poodle | Unspecified | Unspecified |
| Control | Pug | Unspecified dry food + cooked food | Lily’s Kitchen wet food + cooked food |
| Control | Shih Tzu | Cooked food | Orijin + cooked food |

*Legend*: Dietary information from the 6 months preceding examination has been provided where this was available in the clinical notes in patient’s paper records. Commercial names for diets are used where relevant. Where this was not noted, or generic terms were used (“wet”/ “dry” food) the label “unspecified” has been used. In instances where the brand of the diet was recorded, but not the product name, the brand name has been supplied. It should be noted that the same brand name does not equate to the same dietary product. CKCS, Cavalier King Charles Spaniel.

**Supplementary Table 5.** Known comorbidities and medications of dogs in the (a) affected and (b) control populations.

**a.** Affected dogs.

| Breed | Comorbidities (Visit 1) | Comorbidities (Visit 2) | Medication (Visit 1) | Medication (Visit 2) |
| --- | --- | --- | --- | --- |
| CKCS |  |  |  | ACEi, furosemide, pimobendan, spironolactone |
| CKCS |  | Skin disease (unspecified) |  | Furosemide, pimobendan, topical shampoo (2% miconazole nitrate and 2% chlorhexidine gluconate) |
| CKCS |  | Osteoarthritis |  | ACEi, carprofen, furosemide, theophylline |
| CKCS |  |  |  | Furosemide, pimobendan, theophylline |
| CKCS | Skin disease (unspecified) |  | Chlorphenamine | Pimobendan |
| CKCS |  |  |  | ACEi, furosemide, pimobendan, spironolactone |
| CKCS | Skin disease (unspecified), seizures |  |  | Furosemide, pimobendan |
| CKCS | Otitis externa |  | Topical aural drops (miconazole nitrate, polymyxin B, prednisolone) |  |
| CKCS |  |  |  | Furosemide, pimobendan |
| CKCS | Anal gland infection, syringomyelia | Syringomyelia | Carprofen, clindamycin, gabapentin, topical ocular drops (fusidic acid 10 mg, benzalkonium chloride, 0.1 mg, disodium edetate, mannitol, carbomer, sodium hydroxide and water per 1g of solution) | Gabapentin, furosemide, pimobendan, omega 3 supplements |
| CKCS | Gastroenteritis (within 3 months) | Seizures | Corticosteroids (within 3 months), ranitidine (within 3 months) | ACEi, furosemide, gabapentin, meloxicam, phenobarbitone |
| CKCS | Keratoconjunctivitis sicca, dental disease | Keratoconjunctivitis sicca, dental disease | Clindamycin, topical ocular ciclosporin | ACEi, furosemide, pimobendan, spironolactone, topical ocular ciclosporin |
| CKCS |  | Osteoarthritis |  | Furosemide, gabapentin, meloxicam, pimobendan |
| CKCS |  |  |  | Furosemide, pimobendan |
| CKCS |  |  |  | Furosemide, pimobendan |
| CKCS | Conjunctivitis, dermatitis, syringomyelia | Osteoarthritis, syringomyelia | Topical ocular drops (fusidic acid 10 mg, benzalkonium chloride, 0.1 mg, disodium edetate, mannitol, carbomer, sodium hydroxide and water per 1g of solution) | Carprofen, furosemide, gabapentin |
| CKCS |  |  | ACEi | Pimobendan, spironolactone, theophylline |
| CKCS |  |  |  | ACEi, furosemide, pimobendan, spironolactone |
| CKCS |  |  |  | Carprofen |
| CKCS |  |  |  | ACEi, furosemide, pimobendan, spironolactone |
| Bichon Frisé | Osteoarthritis | Osteoarthritis, swollen foot | Glucosamine chondroitin | Glucosamine chondroitin |
| Chihuahua | Dental disease |  |  | ACEi, furosemide, pimobendan |
| Cocker Spaniel | Mammary mass | Diabetes mellitus | ACEi | ACEi, amoxicillin/ clavulanic acid, furosemide, insulin, pimobendan |
| Crossbreed |  |  | Acepromazine, diazepam (both occasional) |  |
| Crossbreed | Chronic gastroenteritis, flea allergic dermatitis | Chronic gastroenteritis | Antibiotics (unspecified), dexamethasone (within 3 months) | Furosemide, pimobendan |
| Crossbreed | Canine infectious respiratory disease, dental disease |  | Clindamycin, doxycycline | ACEi, furosemide, pimobendan |
| Maltese |  | Cough |  | Theophylline |
| Shih Tzu |  |  |  | ACEi, furosemide, pimobendan, theophylline |
| Shih Tzu |  |  |  | Furosemide, pimobendan |
| Yorkshire Terrier | Dental disease, skin disease (unspecified) |  | Cefalexin, topical shampoo (2% miconazole nitrate and 2% chlorhexidine gluconate) | ACEi, furosemide, pimobendan, theophylline |

**b.** Control dogs.

| Breed | Comorbidities (Visit 1) | Comorbidities (Visit 2) | Medication (Visit 1) | Medication (Visit 2) |
| --- | --- | --- | --- | --- |
| CKCS |  | Osteoarthritis |  | Meloxicam |
| CKCS | Keratoconjunctivitis sicca, syringomyelia | Keratoconjunctivitis sicca, syringomyelia | Cimetidine, gabapentin, omega 3 supplements, meloxicam, metronidazole, propentofylline, topical ocular ciclosporin | Gabapentin, omega 3 supplements, topical ocular ciclosporin |
| CKCS |  | Chronic gastroenteritis |  |  |
| CKCS |  |  |  |  |
| CKCS |  |  |  |  |
| Jack Russell Terrier |  |  |  |  |
| Poodle |  | Acute gastroenteritis (within 3 months) |  | Metronidazole (within 3 months) |
| Poodle |  |  |  |  |
| Pug | Osteoarthritis | Osteoarthritis | Meloxicam | Meloxicam |
| Shih Tzu |  | Keratoconjunctivitis sicca, superficial corneal ulcer |  | Topical ocular ciclosporin |

*Legend*: Information from the 6 months preceding examination has been provided where this was available in clinical records. Notes were transcribed into a local data repository by a series of postgraduate students and nurses associated with the clinic. Blank entries may reflect a lack of known comorbidities/ medication or a failure on behalf of owners or clinic staff to report this. ACEi, angiotensin converting enzyme inhibitor; CKCS, Cavalier King Charles Spaniel.

**Supplementary Figure 1.** A volcano-style plot of orthogonal projection to latent structure discriminant analysis (OPLS-DA) model weights against the Q values generated by a linear mixed effects analysis of the association between disease stage and feature intensity

in positive ionisation mode.

**
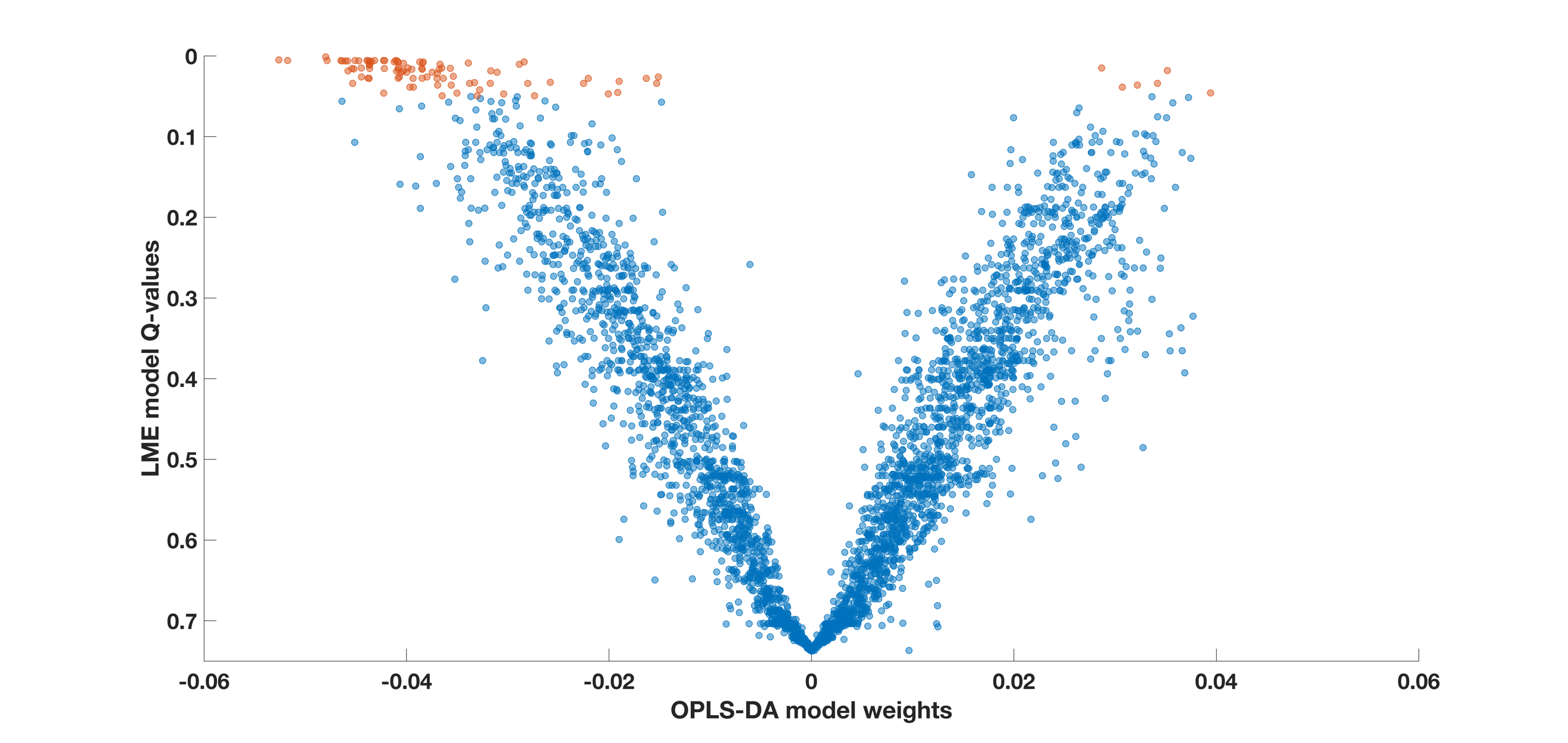
**

*Legend:* Each point represents a feature from positive polarity ionization (lipid RPC+) analysis. Features found to be statistically significant in the linear mixed effects model (Q value < 0.05) are coloured in orange. The x axis represents the weight of disease stage on feature intensity in orthogonal projection to latent structure discriminant analyses (OPLS-DA). The y axis is in descending order and represents the corresponding significance (Q value) from linear mixed effects (LME) modelling where feature intensity was regressed on disease stage.
